# Escore Albumina-Bilirrubina para Predizer Desfechos em Pacientes com Cardiomiopatia Dilatada Idiopática

**DOI:** 10.36660/abc.20210035

**Published:** 2022-06-06

**Authors:** Mei Jiang, Xue-biao Wei, Jie-leng Huang, Ze-da-zhong Su, Ying-wen Lin, Dan-qing Yu

**Affiliations:** 1 Southern Medical University The Second School of Clinical Medicine Guangzhou Guangdong China The Second School of Clinical Medicine, Southern Medical University, Guangzhou, Guangdong – China; 2 Guangdong Provincial People's Hospital Guangdong Cardiovascular Institute Department of Cardiology Guangzhou Guangdong China Department of Cardiology, Guangdong Cardiovascular Institute, Guangdong Provincial People's Hospital, Guangdong Academy of Medical Sciences, Guangzhou, Guangdong – China; 3 Guangdong Provincial People's Hospital Guangdong Provincial Geriatrics Institute Department of Geriatric Intensive Medicine Guangzhou Guangdong China Department of Geriatric Intensive Medicine, Guangdong Provincial Geriatrics Institute, Guangdong Provincial People's Hospital, Guangdong Academy of Medical Sciences, Guangzhou, Guangdong – China

**Keywords:** Cardiomiopatia Dilatada, Insuficiência Cardíaca, Prognóstico

## Abstract

**Fundamento:**

A disfunção hepática é uma variável postulada de prognóstico desfavorável na cardiomiopatia dilatada (CMD).

**Objetivo:**

Este estudo teve como objetivo investigar o valor prognóstico do escore albumina-bilirrubina (ALBI), um modelo relativamente novo para a avaliação da função hepática, em pacientes com CMD idiopática.

**Métodos:**

Um total de 1.025 pacientes com CMD idiopática foram incluídos retrospectivamente e divididos em três grupos com base nos escores de ALBI: grau 1 (≤ −2,60, n = 113), grau 2 (−2,60 a −1,39, n = 835) e grau 3 (> −1,39, n = 77). Foi analisada a associação do escore ALBI com eventos clínicos adversos maiores (ECAM) intra-hospitalares e mortalidade a longo prazo. Valor de p inferior a 0,05 foi considerado estatisticamente significativo.

**Resultados:**

A taxa de ECAM intra-hospitalares foi significativamente maior nos pacientes com grau 3 (2,7% versus 7,1% versus 24,7%, p < 0,001). A análise multivariada mostrou que o escore ALBI foi um preditor independente para ECAM intra-hospitalares (*odds ratio* ajustada = 2,80, IC 95%: 1,63 – 4,80, p < 0,001). Após seguimento mediano de 27 meses, 146 (14,2%) pacientes morreram. A curva de Kaplan-Meier indicou que a taxa cumulativa de sobrevida a longo prazo foi significativamente menor em pacientes com grau mais alto de ALBI (log-rank = 45,50, p < 0,001). O escore ALBI foi independentemente associado à mortalidade a longo prazo (*hazard ratio* ajustada = 2,84, IC 95%: 1,95 – 4,13, p < 0,001).

**Conclusão:**

O escore ALBI, como modelo de risco simples, pode ser considerado uma ferramenta de estratificação de risco para pacientes com CMD idiopática.

## Introdução

A cardiomiopatia dilatada (CMD), uma das principais causas de insuficiência cardíaca, caracteriza-se por dilatação ventricular e disfunção sistólica.^1^ Cerca de 50% dos casos têm causa desconhecida, o que é denominado CMD idiopática.^2^ Dados epidemiológicos indicam que a mortalidade em um ano da CMD é de 25% a 30%, e que aumentou continuamente em 5 anos.^3^ Portanto, a avaliação contínua do risco é essencial para identificar pacientes com alto risco de morte e estabelecer estratégias de tratamento ideais para melhorar o prognóstico.

A lesão hepática é comum em pacientes com insuficiência cardíaca por causa da perfusão prejudicada e da congestão sistêmica devido a alterações hemodinâmicas.^4^ A disfunção hepática foi identificada como um dos fatores de risco para desfechos desfavoráveis em pacientes com CMD.^5^ O escore albumina-bilirrubina (ALBI) é um método simples e objetivo para avaliar a função hepática. Em estudos anteriores, o escore ALBI tem sido amplamente utilizado em pacientes com hepatopatias, incluindo carcinoma hepatocelular, cirrose hepática e insuficiência hepática.^6-8^ Além disso, Matsue e colaboradores indicaram que o escore ALBI está associado à sobrecarga hídrica e ao prognóstico de pacientes com insuficiência cardíaca aguda.^9^ No entanto, ainda não está claro se esse escore pode ser considerado uma ferramenta de estratificação de risco em pacientes com CMD idiopática. Portanto, o presente estudo foi realizado para investigar a associação do escore ALBI e desfechos adversos na CMD idiopática.

## Métodos

### Desenho do estudo e pacientes

Trata-se de um estudo de coorte retrospectivo realizado no Hospital Popular da Província de Guangdong. Pacientes diagnosticados com CMD idiopática foram incluídos consecutivamente entre janeiro de 2010 e novembro de 2015. O diagnóstico de CMD estava de acordo com a declaração do grupo de trabalho de doenças miocárdicas e pericárdicas da Sociedade Europeia de Cardiologia.^10^ Os critérios de exclusão foram os seguintes: 1) idade < 18 anos; 2) presença de tumor maligno; 3) gravidez; 4) doença autoimune; 5) histórico de terapia de sincronização cardíaca ou transplante cardíaco; e 6) CMD com etiologia definida, como cardiopatia hipertensiva, doença arterial coronariana (> 50% de lesão obstrutiva em um ou mais vasos epicárdicos), valvopatia, cardiopatia congênita, desencadeantes de miocardite, cardiomiopatia alcoólica, cardiomiopatia periparto, cardiomiopatia causada por distúrbio endócrino, não compactação do miocárdio ventricular e cardiomiopatia induzida por arritmia. Além disso, também excluímos pacientes sem registros de albumina ou bilirrubina sérica de admissão. Foram incluídos um total de 1.025 pacientes com CMD idiopática. O presente estudo foi aprovado pelo comitê de ética do Hospital Popular da Província de Guangdong, com dispensa de consentimento informado.

### Examinação e coleta de dados

Foram coletadas amostras de sangue venoso para medir as concentrações de albumina e bilirrubina pela manhã após pernoite. Os níveis séricos de albumina e bilirrubina foram detectados em um analisador bioquímico automatizado (Beckman Coulter AU5821 ou AU5831; Beckman Coulter Inc, Califórnia, EUA). O ecocardiograma transtorácico foi realizado rotineiramente dentro de 24 horas da admissão. Foram medidos diâmetro do átrio esquerdo (DAE), diâmetro diastólico final do ventrículo esquerdo (DDFVE), fração de ejeção do ventrículo esquerdo (FEVE) e outros índices de ecocardiograma de acordo com as recomendações da Sociedade Americana de Ecocardiografia.^11^

As variáveis clínicas foram coletadas do prontuário eletrônico por um pesquisador e verificadas aleatoriamente por outro. A taxa de filtração glomerular estimada (TFGe, expressa em mL/min/1,73 m^2^) foi calculada usando a equação Chronic Kidney Disease Epidemiology Collaboration equation.^12^ O escore ALBI foi calculado usando a fórmula seguinte: (0,66× log_10_ bilirrubina-0,085×albumina).

### Acompanhamento e desfechos

Todos os pacientes com sobrevida intra-hospitalar foram acompanhados por meio de entrevistas telefônicas. Também revisamos os registros de readmissão hospitalar e entrevistas ambulatoriais para possíveis eventos. O desfecho primário foi mortalidade a longo prazo e o desfecho secundário compreendeu eventos clínicos adversos maiores (ECAM) intra-hospitalares, como morte, acidente vascular cerebral, diálise e insuficiência cardíaca aguda durante a hospitalização.

### Análise estatística

Os pacientes incluídos foram divididos em 3 grupos com base na pontuação ALBI: grau 1 (≤ −2,60, n = 113); grau 2 (−2,60 a −1,39, n = 835); e grau 3 (> −1,39, n = 77). A distribuição das variáveis foi avaliada pelo teste de Kolmogorov-Smirnov. As variáveis contínuas com distribuição normal são apresentadas como média ± desvio padrão e as variáveis contínuas com distribuição não normal são apresentadas como mediana e intervalo interquartil. As variáveis categóricas são apresentadas em números e percentuais. As variáveis contínuas foram comparadas usando ANOVA de uma via quando normalmente distribuídas e o teste Kruskal-Wallis H quando não normalmente distribuídas. O teste do qui-quadrado foi realizado para as variáveis categóricas. A análise da curva característica de operação do receptor (ROC) foi usada para determinar os níveis de corte ideais do escore ALBI para predizer eventos adversos. Foram usadas a regressão logística e a análise de sobrevivência de Cox para avaliar o efeito do escore ALBI em ECAM intra-hospitalares e mortalidade a longo prazo, respectivamente. Variáveis significativas na análise univariada (exceto os elementos do ALBI) foram incluídas na análise multivariada. Além disso, curvas de Kaplan-Meier foram elaboradas e comparadas pelo teste log-rank entre os grupos. Para todas as análises, p < 0,05 foi considerado como indicativo de significância estatística. Todas as análises foram realizadas no software SPSS (versão 16,0; SPSS Inc, Chicago, Illinois, EUA).

## Resultados

Em total, foram incluídos 1.025 pacientes nesta análise. As características basais entre os grupos são apresentadas na Tabela 1. Os pacientes no grupo com grau 3 eram mais propensos a serem masculinos. Além disso, pacientes com grau maior de ALBI apresentaram pior função cardíaca, isto é, foi maior a taxa de pacientes com classe funcional da New York Heart Association (NYHA) > II. Foram observadas tendências positivas para creatinina sérica, alanina transaminase (ALT), bilirrubina total e DAE em relação ao aumento do escore de ALBI. No entanto, observou-se tendência negativa para hemoglobina e albumina sérica em relação ao aumento do escore ALBI. Diuréticos (incluindo furosemida e espironolactona) e digoxina foram usados com mais frequência em pacientes com grau mais alto de ALBI.

**Tabela 1:** Características basais classificadas por tercil de grau de ALBI

Variáveis clínicas		Grau 1 (n=113)	Grau 2 (n=835)	Grau 3 (n=77)	p
**Idade (anos)**		52,8±12,5	55,9±13,6	52,7±16,2	0,018
**Sexo**					
	Masculino, n (%)	70(61,9)	609(72,9)	65(84,4)	0,003
	Feminino, n (%)	43(38,1)	226(27,1)	12(15,6)	
**Hipertensão, n (%)**		31(27,4)	221(26,5)	18(23,4)	0,809
**Diabetes, n (%)**		15(13,3)	148(17,7)	9(11,7)	0,228
**Tabagismo, n (%)**		29(25,7)	233(27,9)	20(26,0)	0,840
**Classe funcional da NYHA>II**		43(38,1)	445(53,3)	53(68,8)	<0,001
**Hemoglobina (g/L)**		143,3±17,0	139,4±18,4	134,0±24,6	0,004
**Creatinina sérica, (umol/L)**		85,0(69,3,102,5)	94,0(78,5,113,0)	113,5(90,0,152,0)	<0,001
**Testes de função hepática**					
	ALT (U/L)	24,5(16,8,34,0)	29,0(19,0,48,0)	31,5(20,3,106,8)	0,001
	Albumina (g/L)	41,9±2,2	34,8±3,5	25,9±3,3	<0,001
**Bilirrubina total, (μmol/L)**		15,6(11,4,20,8)	21,6(15,1,31,2)	45,7(23,7,78,3)	<0,001
**Dados ecocardiográficos**					
	DAE, (mm)	41,4±7,0	44,6±7,2	46,8±9,5	<0,001
	DDFVE, (mm)	67,1±8,3	67,0±8,7	68,0±8,0	0,604
	FEVE, (%)	30,1±7,5	29,2±7,7	27,5±8,7	0,075
**Medicamentos durante a hospitalização**					
	IECA/BRA	95(84,1)	708(84,8)	60(77,9)	0,286
	Betabloqueadores	90(79,6)	658(78,8)	54(70,1)	0,196
	Lasix	90(79,6)	730(87,4)	72(93,5)	0,015
	Aldactone	89(78,8)	741(88,7)	72(93,5)	0,003
	Digoxina	48(42,5)	509(61,0)	65(84,4)	<0,001
	ECAM intra-hospitalares	3(2,7)	59(7,1)	19(24,7)	<0,001

ALBI: albumina-bilirrubina; ALT: alanina transaminase; BRA: bloqueador do receptor de angiotensina; DAE: diâmetro do átrio esquerdo; DDFVE: dimensão diastólica final do ventrículo esquerdo; ECAM: eventos cardíacos adversos maiores; FEVE: fração de ejeção do ventrículo esquerdo; IECA: inibidores da enzima conversora de angiotensina; NYHA: New York Heart Association.

Durante a internação hospitalar, 15 pacientes (1,5%) foram a óbito; 48 (4,7%) apresentaram insuficiência cardíaca aguda; 23 (2,2%) necessitaram de diálise renal e 23 (2,2%) apresentaram acidente vascular cerebral. A taxa de ECAM intra-hospitalar foi significativamente maior em pacientes com grau 3 do que naqueles com graus 1 e 2 (2,7% versus 7,1% versus 24,7%, p < 0,001, Tabela 1). Na análise de regressão logística univariada, escore ALBI, classe funcional da NYHA > II, anemia, TFGe < 60 mL/min/1,73 = m^2^, lgALT(log^10^ALT), bilirrubina total, DAE, DDFVE, FEVE, e uso de betabloqueador foram associados aos ECAM intra-hospitalares (Tabela 2). Após ajuste para potenciais fatores de risco, o escore ALBI foi um preditor independente de ECAM intra-hospitalares (*odds ratio* ajustada = 2,80, intervalo de confiança [IC] de 95%: 1,63 – 4,80, p < 0,001, Tabela 2).

**Tabela 2:** Análise de regressão logística univariada e multivariada para para ECAM intra-hospitalares

Variáveis clínicas	Análise univariada			Análise multivariada	
	OR	p	OR	95% IC	p
Escore ALBI	4,07	<0,001	2,80	1,63 – 4,80	<0,001
Idade (anos)	1,01	0,440			
Sexo feminino	0,92	0,754			
Hipertensão	0,85	0,540			
Diabetes	1,24	0,456			
Tabagismo	0,85	0,554			
Classe funcional da NYHA>II	1,88	0,010	1,20	0,70 – 2,05	0,506
Anemia	2,16	0,015	1,75	0,88 – 3,47	0,112
TFGe<60mL/min/1,73 m^2^	2,42	<0,001	1,70	1,02 – 2,83	0,040
lgALT	2,73	<0,001	1,77	1,08 – 2,92	0,025
Hipoproteinemia	2,48	<0,001			
Bilirrubina total	1,01	0,001			
DAE	1,03	0,049	1,01	0,97 – 1,04	0,680
DDFVE	1,04	0,004	1,03	1,00 – 1,06	0,085
FEVE	0,95	0,001	0,97	0,94 – 1,01	0,152
Uso de IECA/BRA	0,74	0,312			
Uso de betabloqueador	0,41	<0,001	0,47	0,28 – 0,79	0,004
Uso de lasix	1,21	0,603			
Uso de aldactone	0,97	0,921			
Uso de digoxina	1,59	0,065			

ALBI: albumina-bilirrubina; ALT: alanina transaminase; BRA: bloqueador do receptor de angiotensina; BT: bilirrubina total; DAE: diâmetro do átrio esquerdo; DB: direct bilirubin; DDFVE: dimensão diastólica final do ventrículo esquerdo; FEVE: fração de ejeção do ventrículo esquerdo; IC: intervalo de confiança; IECA: inibidores da enzima conversora de angiotensina; NYHA: New York Heart Association; OR: odds ratio; TFGe: taxa de filtração glomerular estimada.

**Figura 1:**
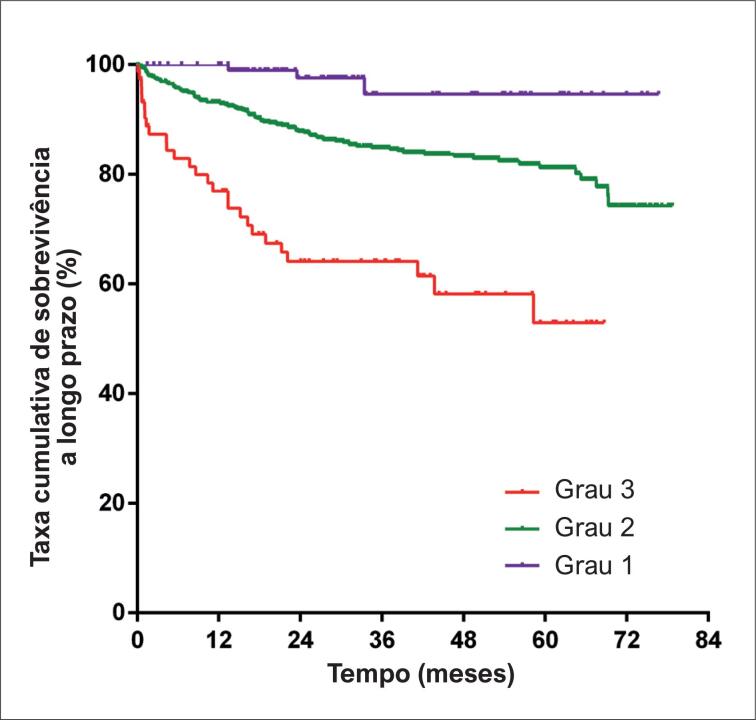
Curva Kaplan–Meier de sobrevida geral.

Após seguimento mediano de 27 meses, 146 (14,2%) pacientes foram a óbito. A curva de Kaplan-Meier indicou que a taxa cumulativa de sobrevida a longo prazo foi significativamente menor em pacientes com grau mais alto de ALBI (teste log-rank = 45,50, p < 0,001, Figura 1). O modelo de risco proporcional univariado de Cox de mortalidade a longo prazo é mostrado na Tabela 3. O escore ALBI foi associado com risco aumentado de morte a longo prazo (*hazard ratio* não ajustada = 3,16, IC 95%: 2,31 – 4,33, p < 0,001). Outras variáveis significativas incluíram idade, classe funcional da NYHA, anemia, TFGe < 60 mL/min/1,73 m^2^, lgALT, hipoproteinemia, bilirrubina total, DAE, DDFVE, FEVE, e uso de betabloqueador e digoxina. Esses fatores de risco significativos, exceto os componentes do escore ALBI, foram incluídos no modelo multivariado de sobrevivência de Cox, que revelou que o escore ALBI permaneceu um preditor independente para mortalidade a longo prazo (*hazard ratio* ajustada = 2,84, IC 95%: 1,95 – 4,13, p < 0,001, Tabela 4). Além disso, o escore ALBI foi incluído neste modelo como uma variável categórica e não contínua. O resultado mostrou que, em comparação com grau 1 de ALBI, a *hazard ratio* ajustada foi de 5,69 (IC 95%: 1,40 – 23,18, p = 0,015, Tabela 4) e 16,79 (IC 95%: 3,91 – 72,04, p < 0,001, Tabela 4) para grau 2 e 3, respectivamente.

**Tabela 3:** Risco proporcional univariado de Cox de mortalidade a longo prazo

Variáveis clínicas	HR	IC 95%	p
Escore ALBI	3,16	2,31 – 4,33	<0,001
Idade (anos)	1,03	1,02 – 1,04	<0,001
Sexo feminino	0,96	0,67 – 1,39	0,845
Hipertensão	0,99	0,69 – 1,44	0,975
Diabetes	0,96	0,62 – 1,49	0,854
Tabagismo	1,03	0,72 – 1,49	0,859
Classe funcional da NYHA>II	1,81	1,28 – 2,54	0,001
Anemia	1,97	1,25 – 3,10	0,003
TFGe<60mL/min/1,73 m^2^	2,09	1,51 – 2,91	<0,001
lgALT	1,78	1,21 – 2,62	0,004
Hipoproteinemia	2,46	1,73 – 3,48	<0,001
Bilirrubina total	1,01	1,00 – 1,01	<0,001
DAE	1,03	1,01 – 1,05	0,016
DDFVE	1,04	1,03 – 1,06	<0,001
FEVE	0,96	0,94 – 0,98	<0,001
Uso de IECA/BRA	0,93	0,60 – 1,44	0,733
Uso de betabloqueador	0,53	0,37 – 0,75	<0,001
Uso de lasix	1,08	0,67 – 1,76	0,742
Uso de aldactone	1,43	0,83 – 2,48	0,202
Uso de digoxina	1,55	1,09 – 2,20	0,016

ALBI: albumina-bilirrubina; ALT: alanina transaminase; BRA: bloqueador do receptor de angiotensina; DAE: diâmetro do átrio esquerdo; DDFVE: dimensão diastólica final do ventrículo esquerdo; FEVE: fração de ejeção do ventrículo esquerdo; HR: hazard ratio; IC: intervalo de confiança; IECA: inibidores da enzima conversora de angiotensina; NYHA: New York Heart Association; TFGe: taxa de filtração glomerular estimada.

**Tabela 4:** Risco proporcional multivariado de Cox de mortalidade a longo prazo

Variáveis clínicas		HR	IC 95%	p
**Modelo 1**				
ALBI		2,84	1,95 – 4,13	<0,001
Idade (anos)		1,03	1,02 – 1,05	<0,001
Classe funcional da NYHA>II		1,25	0,86 – 1,82	0,236
Anemia		1,25	0,76 – 2,06	0,382
TFGe<60mL/min/1,73 m^2^		1,30	0,91 – 1,85	0,156
lgALT		1,46	1,00 – 2,14	0,050
DAE		1,00	0,98 – 1,03	0,898
DDFVE		1,04	1,02 – 1,06	<0,001
FEVE		0,99	0,97 – 1,01	0,348
Uso de betabloqueador		0,65	0,45 – 0,95	0,024
Uso de digoxina		1,05	0,72 – 1,54	0,804
**Modelo 2**				
ALBI				
	Grau 1	-	-	-
	Grau 2	5,69	1,40 – 23,18	0,015
	Grau 3	16,79	3,91 – 72,04	<0,001
Idade (anos)		1,03	1,02 – 1,05	<0,001
Classe funcional da NYHA>II		1,24	0,85 – 1,81	0,262
Anemia		1,37	0,84 – 2,24	0,205
TFGe<60mL/min/1,73 m^2^		1,29	0,90 – 1,84	0,168
lgALT		1,57	1,08 – 2,28	0,019
DAE		1,00	0,98 – 1,03	0,758
DDFVE		1,04	1,02 – 1,07	<0,001
FEVE		0,98	0,96 – 1,01	0,180
Uso de betabloqueador		0,59	0,41 – 0,85	0,005
Uso de digoxina		1,08	0,74 – 1,58	0,702

ALBI: albumina-bilirrubina; ALT: alanina transaminase; DAE: diâmetro do átrio esquerdo; DDFVE: dimensão diastólica final do ventrículo esquerdo; FEVE: fração de ejeção do ventrículo esquerdo; HR: hazard ratio; IC: intervalo de confiança; NYHA: New York Heart Association; TFGe: taxa de filtração

**Figura 2:**
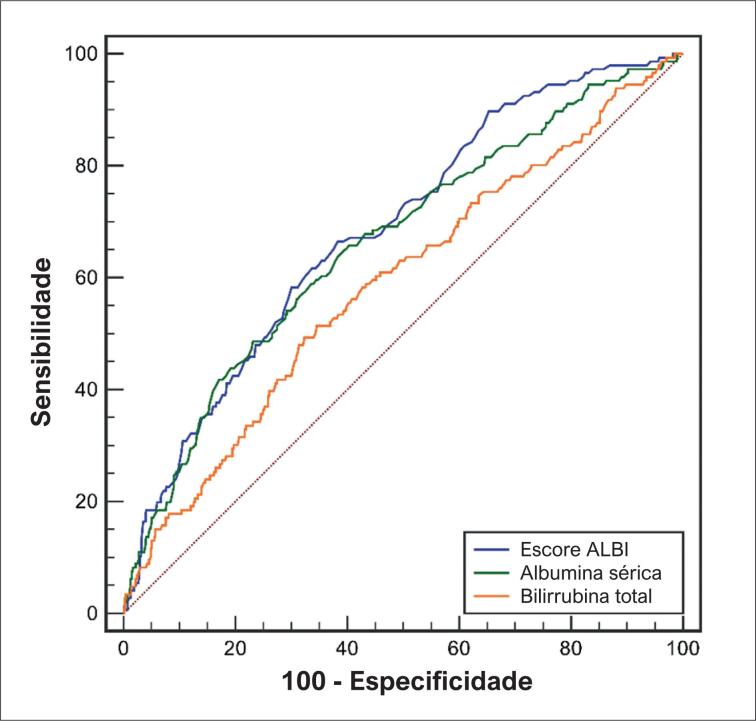
Análise ROC de mortalidade a longo prazo.

A análise da curva ROC indicou que a área sob a curva do escore ALBI, albumina sérica e bilirrubina total para predizer a morte a longo prazo foi de 0,684 (IC 95%: 0,654 – 0,714, Figura 2), 0,662 (IC 95%: 0,631 – 0,692, Figura 2) e 0,588 (IC 95%: 0,556 – 0,619, Figura 2), respectivamente. O escore ALBI demonstrou capacidade preditiva relativamente superior para morte a longo prazo do que a albumina sérica (0,684 versus 0,662, p = 0,026, Figura 2) e bilirrubina total (0,684 versus 0,588, p = 0,002, Figura 2).

## Discussão

Até onde sabemos, este é o primeiro estudo a avaliar o papel prognóstico do escore ALBI em pacientes com CMD idiopática. Os resultados mostraram que o escore ALBI foi um fator de risco independente para ECAM intra-hospitalares e mortalidade a longo prazo. Além disso, o escore ALBI apresentou melhor capacidade preditiva para morte a longo prazo do que a albumina sérica e a bilirrubina total. O escore ALBI pode ser facilmente medido e seria útil na identificação de pacientes com CMD idiopática que apresentam alto risco de desfechos desfavoráveis.

A CMD é caracterizada por remodelação ventricular que pode evoluir gradualmente para insuficiência cardíaca esquerda e até insuficiência cardíaca global.^13,14^ Além disso, a disfunção ventricular direita é prevalente em pacientes com CMD,^15^ e tem demonstrado influenciar o curso e o prognóstico da CMD.^16^ A progressão da disfunção ventricular direita pode levar à congestão sistêmica, resultando em congestão sinusoidal e edema perissinusoidal, que prejudicam o fornecimento de oxigênio e nutrientes aos hepatócitos.^17-19^ Além disso, o débito cardíaco diminuído e a perfusão hepática inadequada podem desencadear lesão hipóxica. Essa lesão dos hepatócitos pode se manifestar como albumina sérica diminuída e bilirrubina elevada.

A albumina, que reflete a função sintética do fígado, tem múltiplos papéis fisiológicos, como contrabalançar a pressão hidrostática, funções antioxidantes e anti-inflamatórias e transportar moléculas e drogas.^20^ Verificamos que a hipoalbuminemia estava relacionada a resultados adversos em pacientes com CMD idiopática. Isso pode ser explicado por várias teorias. Em primeiro lugar, além de ser um marcador de lesão hepática, a hipoalbuminemia está frequentemente associada à disfunção renal.^20,21^ A albumina é restringida pela barreira glomerular normal, e a albumina filtrada pode ser reabsorvida pelas células tubulares proximais.^22^ No entanto, o aumento da secreção urinária de proteína pode ser encontrada na insuficiência renal, que resulta em hipoalbuminemia. Portanto, a hipoalbuminemia pode refletir a disfunção renal concomitante e pressagiar desfechos desfavoráveis. Em segundo lugar, a hipoalbuminemia resulta em pressão osmótica sérica mais baixa e pode exacerbar o edema pulmonar e o derrame pleural, precipitando insuficiência cardíaca refratária em pacientes com CMD.^21^ Em terceiro lugar, tem sido demonstrado que os níveis séricos de albumina e pré-albumina refletem o estado nutricional.^23,24^ A má-nutrição às vezes pode evoluir para caquexia cardíaca, que é caracterizada por desnutrição protéico-calórica com perda muscular e edema periférico, levando a pior qualidade de vida e aumento da mortalidade.^24^

De forma semelhante, em pacientes com CMD avançada, vários processos metabólicos da bilirrubina no fígado, incluindo captação, conjugação e secreção, são atenuados pela hipóxia e a congestão hepatocelular, levando a um aumento maior da bilirrubina sérica total. Embora a bilirrubina tenha propriedades antioxidantes e anti-inflamatórias, níveis extremamente elevados de bilirrubina representam comprometimento hemodinâmico causado por disfunção ventricular direita, que tem um efeito prognóstico adverso em pacientes com CMD.^16^ Além disso, a hiperbilirrubinemia reflete um estado cardíaco adverso latente na insuficiência cardíaca crônica.^25^ Lang et al. indicaram que a bilirrubina tem efeitos adversos nos eritrócitos, induzindo eriptose. O dano excessivo aos eritrócitos leva à anemia grave e afeta ainda mais o prognóstico.^26^ Essas evidências corroboram o nosso achado de que a hiperbilirrubinemia é um fator de risco para pacientes com CMD idiopática.

Tanto a hipoalbuminemia quanto a hiperbilirrubinemia foram fatores de risco para o prognóstico adverso em pacientes com CMD idiopática. O escore ALBI, combinando esses dois efeitos, tem sido amplamente testado como um método objetivo, simples e diferenciador para avaliar a função hepática.^27^ Até onde sabemos, nenhum estudo tem avaliado o valor prognóstico do escore ALBI em pacientes com CMD. O presente estudo demonstrou que o escore ALBI foi independentemente associado a desfechos adversos intra-hospitalares e de longo prazo. O escore ALBI consiste em apenas duas variáveis e é uma ferramenta simples de estratificação de risco em pacientes com CMD idiopática. Com base no presente estudo, a aplicação clínica do escore ALBI pode ser estendida de doenças hepáticas para a CMD idiopática.

### Limitações

Nosso estudo tem algumas limitações. Em primeiro lugar, este foi um estudo de coorte retrospectivo; portanto, alguns níveis de bilirrubina e albumina de admissão estavam ausentes, o que pode afetar os resultados. Em segundo lugar, a bilirrubina e a albumina não foram detectadas dinamicamente. A relação entre o prognóstico e o escore ALBI em diferentes momentos é desconhecida. Finalmente, como a nossa população de estudo incluída não representava pacientes com CMD idiopática em contextos diversos, por exemplo, em países ocidentais, os resultados do estudo devem ser validados em diferentes coortes com CMD idiopática.

## Conclusões

O presente estudo demonstrou que o escore ALBI foi independentemente associado ao aumento do risco de ECAM intra-hospitalares e mortalidade a longo prazo em pacientes com CMD idiopática. Além disso, comparado à bilirrubina e à albumina, o escore ALBI apresentou capacidade preditiva relativamente superior para mortalidade a longo prazo, que pode identificar mais pacientes com alto risco de desfechos desfavoráveis.
