# Supplementary material for: Escore Albumina-Bilirrubina para Predizer Desfechos em Pacientes com Cardiomiopatia Dilatada Idiopática
Source: Arq Bras Cardiol. 2022 Jun 6;118(6):1108–15. [Article in Portuguese] doi: 10.36660/abc.20210035 (PMC9345152; doi:10.36660/abc.20210035)
Supplement: Translation

# Translation: Albumin-Bilirubin Score to Predict Outcomes in Patients with Idiopathic Dilated Cardiomyopathy

**Authors:** Mei Jiang, Xue-biao Wei, Jie-leng Huang, Ze-da-zhong Su, Ying-wen Lin, Dan-qing Yu

**Affiliations:** 1 Guangzhou Guangdong China The Second School of Clinical Medicine, Southern Medical University, Guangzhou, Guangdong – China; 2 Guangzhou Guangdong China Department of Cardiology, Guangdong Cardiovascular Institute, Guangdong Provincial People's Hospital, Guangdong Academy of Medical Sciences, Guangzhou, Guangdong – China; 3 Guangzhou Guangdong China Department of Geriatric Intensive Medicine, Guangdong Provincial Geriatrics Institute, Guangdong Provincial People's Hospital, Guangdong Academy of Medical Sciences, Guangzhou, Guangdong – China

**Keywords:** Dilated Cardiomyopathy, Heart Failure, Prognosis

## Abstract

**Background:**

Liver dysfunction is a postulated variable for poor prognosis in dilated cardiomyopathy (DCM).

**Objective:**

This study aimed to investigate the prognostic value of the albumin-bilirubin (ALBI) score, a relatively new model for evaluating liver function, in patients with idiopathic DCM.

**Methods:**

A total of 1025 patients with idiopathic DCM were retrospectively included and divided into three groups based on ALBI scores: grade 1 (≤ −2.60, n = 113), grade 2 (−2.60 to −1.39, n = 835), and grade 3 (> −1.39, n = 77). The association of ALBI score with in-hospital major adverse clinical events (MACEs) and long-term mortality was analyzed. P-value less than 0.05 was considered statistically significant.

**Results:**

The in-hospital MACEs rate was significantly higher in the grade 3 patients (2.7% versus 7.1% versus 24.7%, p < 0.001). Multivariate analysis showed that ALBI score was an independent predictor for in-hospital MACEs (adjusted odds ratio = 2.80, 95%CI: 1.63 – 4.80, p < 0.001). After a median 27-month follow-up, 146 (14.2%) patients died. The Kaplan–Meier curve indicated that the cumulative rate of long-term survival was significantly lower in patients with higher ALBI grade (log-rank = 45.50, p < 0.001). ALBI score was independently associated with long-term mortality (adjusted hazard ratio = 2.84, 95%CI: 1.95 – 4.13, p < 0.001).

**Conclusion:**

ALBI score as a simple risk model could be considered a risk-stratifying tool for patients with idiopathic DCM.

## Introduction

Dilated cardiomyopathy (DCM), one of the leading causes of heart failure, is characterized by ventricular dilation and systolic dysfunction.^1^ About 50% of the cases have an unknown cause, which is termed as idiopathic DCM.^2^ Epidemiological data have indicated that the one-year mortality of DCM is 25% to 30%, which continuously increased at 5 years.^3^ Therefore, continued risk assessment is essential to identify patients at high risk of death and establish optimal treatment strategies to improve prognosis.

Liver injury is common in patients with heart failure owing to impaired perfusion and systemic congestion due to hemodynamic changes.^4^ Hepatic dysfunction has been identified as one of the risk factors for poor outcomes in patients with DCM.^5^ The albumin-bilirubin (ALBI) score is a simple and objective method to assess liver function. In previous studies, ALBI score has been widely used in patients with liver diseases, including hepatocellular carcinoma, liver cirrhosis, and liver failure.^6-8^ In addition, Matsue et al indicated that the ALBI score is associated with fluid overload and the prognosis of patients with acute heart failure.^9^ However, it is yet unclear whether this score can be considered a risk-stratifying tool in patients with idiopathic DCM. Hence, this study was conducted to investigate the association of the ALBI score and adverse outcomes in idiopathic DCM.

## Methods

### Study design and patients

This was a retrospective cohort study conducted at Guangdong Provincial People's Hospital. Patients diagnosed with idiopathic DCM were consecutively enrolled between January 2010 and November 2015. The diagnosis of DCM was in agreement with the statement of the European Society of Cardiology working group on myocardial and pericardial diseases.^10^ The exclusion criteria were as follows: 1) age < 18 years; 2) presence of malignant tumor; 3) pregnancy; 4) autoimmune disease; 5) previous cardiac synchronization therapy or heart transplantation; and 6) DCM with definite etiology such as hypertensive heart disease, coronary artery disease (> 50% obstructive lesion in one or more epicardial vessels), valvular heart disease, congenital heart disease, myocarditis triggers, alcoholic cardiomyopathy, peripartum cardiomyopathy, cardiomyopathy caused by endocrine disorder, noncompaction of the ventricular myocardium, and arrhythmia-induced cardiomyopathy. Furthermore, we also excluded patients without admission serum albumin or bilirubin records. A total of 1025 idiopathic DCM patients were enrolled. The present study was approved by the ethics committee of Guangdong Provincial People's Hospital, with a waiver of informed consent.

### Examination and data collection

Venous blood samples were collected for measurement of albumin and bilirubin concentrations in the morning after an overnight stay. Serum albumin and bilirubin levels were detected on an automated biochemical analyzer (Beckman Coulter AU5821 or AU5831; Beckman Coulter Inc, CA, USA). Transthoracic echocardiography was routinely performed within 24 hours of admission. Left atrial diameter (LAD), left ventricular end diastolic diameter (LVEDD), left ventricular ejection fraction (LVEF), and other echocardiogram indices were measured according to the recommendations of the American Society of Echocardiography.^11^

Clinical variables were collected from the electronic case report form by one researcher and randomly checked by another. Estimated glomerular filtration rate (eGFR, expressed in mL/min/1.73 m^2^) was calculated using the Chronic Kidney Disease Epidemiology Collaboration equation.^12^ The ALBI score was calculated using the following formula: 0.66× log_10_ bilirubin-0.085×albumin.^6^

### Follow-up and outcomes

All in-hospital survival patients were followed up through telephone interviews. We also reviewed hospital readmission records and outpatient clinic interviews for possible events. The primary outcome was long-term mortality, and the secondary outcome was in-hospital major adverse clinical events (MACEs) such as death, stroke, dialysis, and acute heart failure during hospitalization.

### Statistical analysis

Included patients were divided into three groups based on ALBI score: grade 1 (≤ −2.60, n = 113); grade 2 (−2.60 to −1.39, n = 835); and grade 3 (> −1.39, n = 77). The distribution of variables was assessed by the Kolmogorov-Smirnov test. Normally distributed continuous variables are presented as mean ± standard deviation, and non-normally distributed continuous variables are presented as median and interquartile range. Categorical variables are presented as numbers and percentage. Continuous variables were compared using one-way ANOVA when normally distributed or the Kruskal-Wallis H test when not normally distributed. The chi-square test was performed for categorical variables. Receiver operating characteristic (ROC) curve analysis was used to determine the optimum cut-off levels of ALBI score for predicting adverse events. Logistic regression and Cox survival analysis were used to assess the effect of ALBI score on in-hospital MACEs and long-term mortality, respectively. Significant variables in univariate analysis (except the elements of ALBI) were included into the multivariate analysis. In addition, Kaplan–Meier curves were drawn and compared using the log-rank test among groups. For all analyses, p < 0.05 was considered to indicate statistical significance. All analyses were conducted using SPSS software (version 16.0; SPSS Inc, Chicago, IL, USA).

## Results

In all, 1025 patients were included in this analysis. Baseline characteristics among the groups are displayed in Table 1. Patients in the grade 3 group were more likely to be male. In addition, patients with higher ALBI grade had worse cardiac function; namely, the rate of patients with New York Heart Association (NYHA) functional class > II was higher. Positive trends were observed for serum creatinine, alanine transaminase (ALT), total bilirubin, and LAD in relation to increasing ALBI score. However, a negative trend was observed for hemoglobin and serum albumin in relation to increasing ALBI score. Diuretics (including furosemide and spironolactone) and digoxin were more frequently used in patients with higher ALBI grade.

**Table 1:** Baseline characteristics classified by tertile of ALBI grade

Clinical variables		Grade 1 (n=113)	Grade 2 (n=835)	Grade 3 (n=77)	p
**Age (years)**		52.8±12.5	55.9±13.6	52.7±16.2	0.018
**Sex**					
	Male, n (%)	70(61.9)	609(72.9)	65(84.4)	0.003
	Female, n (%)	43(38.1)	226(27.1)	12(15.6)	
**Hypertension, n (%)**		31(27.4)	221(26.5)	18(23.4)	0.809
**Diabetes, n (%)**		15(13.3)	148(17.7)	9(11.7)	0.228
**Smokers, n (%)**		29(25.7)	233(27.9)	20(26.0)	0.840
**NYHA functional class>II**		43(38.1)	445(53.3)	53(68.8)	<0.001
**Hemoglobin (g/L)**		143.3±17.0	139.4±18.4	134.0±24.6	0.004
**Serum creatinine, (μmol/L)**		85.0(69.3.102.5)	94.0(78.5.113.0)	113.5(90.0.152.0)	<0.001
**Liver function tests**					
	ALT (U/L)	24.5(16.8.34.0)	29.0(19.0.48.0)	31.5(20.3.106.8)	0.001
	Albumin (g/L)	41.9±2.2	34.8±3.5	25.9±3.3	<0.001
**Total bilirubin, (μmol/L)**		15.6(11.4.20.8)	21.6(15.1.31.2)	45.7(23.7.78.3)	<0.001
**Echocardiography data**					
	LAD, (mm)	41.4±7.0	44.6±7.2	46.8±9.5	<0.001
	LVEDD, (mm)	67.1±8.3	67.0±8.7	68.0±8.0	0.604
	LVEF, (%)	30.1±7.5	29.2±7.7	27.5±8.7	0.075
**Medicine during hospitalization**					
	ACEI/ARB	95(84.1)	708(84.8)	60(77.9)	0.286
	Beta-blockers	90(79.6)	658(78.8)	54(70.1)	0.196
	Lasix	90(79.6)	730(87.4)	72(93.5)	0.015
	Aldactone	89(78.8)	741(88.7)	72(93.5)	0.003
	Digoxin	48(42.5)	509(61.0)	65(84.4)	<0.001
	In-hospital MACEs	3(2.7)	59(7.1)	19(24.7)	<0.001

ACEI: angiotensin-converting enzyme inhibitors; ALBI: albumin-bilirubin; ALT: alanine transaminase; ARB: angiotensin receptor blocker; LAD: left atrial diameter; LVEDD: left ventricular end-diastolic dimension; LVEF: left ventricular ejection fraction; MACEs: major adverse cardiac events; NYHA: New York Heart Association.

During hospitalization, 15 patients (1.5%) died; 48 (4.7%) suffered from acute heart failure; 23 (2.2%) required renal dialysis, and 23 (2.2%) suffered a stroke. The in-hospital MACE rate was significantly higher in patients with grade 3 than in those with grades 1 and 2 (2.7% versus 7.1% versus 24.7%, p < 0.001, Table 1). In univariate logistic regression analysis, ALBI score, NYHA functional class > II, anemia, eGFR < 60 mL/min/1.73 m^2^, lgALT(log_10_ALT), total bilirubin, LAD, LVEDD, LVEF, and β-blocker usage were associated with in-hospital MACEs (Table 2). After adjusting for potential risk factors, ALBI score was an independent predictor of in-hospital MACEs (adjusted odds ratio = 2.80, 95% confidence interval [CI]: 1.63 – 4.80, p < 0.001, Table 2).

**Table 2:** Univariate and multivariable logistic regression analysis for in-hospital MACEs

Clinical variables	Univariate analysis			Multivariate analysis	
	OR	p	OR	95% CI	p
ALBI score	4.07	<0.001	2.80	1.63 – 4.80	<0.001
Age (years)	1.01	0.440			
Female sex	0.92	0.754			
Hypertension	0.85	0.540			
Diabetes	1.24	0.456			
Smokers	0.85	0.554			
NYHA functional class>II	1.88	0.010	1.20	0.70 – 2.05	0.506
Anemia	2.16	0.015	1.75	0.88 – 3.47	0.112
eGFR<60mL/min/1.73 m^2^	2.42	<0.001	1.70	1.02 – 2.83	0.040
lgALT	2.73	<0.001	1.77	1.08 – 2.92	0.025
Hypoproteinemia	2.48	<0.001			
Total bilirubin	1.01	0.001			
LAD	1.03	0.049	1.01	0.97 – 1.04	0.680
LVEDD	1.04	0.004	1.03	1.00 – 1.06	0.085
LVEF	0.95	0.001	0.97	0.94 – 1.01	0.152
ACEI/ARB usage	0.74	0.312
Beta-blocker usage	0.41	<0.001	0.47	0.28 – 0.79	0.004
Lasix usage	1.21	0.603			
Aldactone usage	0.97	0.921			
Digoxin usage	1.59	0.065			

ACEI: angiotensin-converting enzyme inhibitors; ALBI: albumin-bilirubin; ALT: alanine transaminase; ARB: angiotensin receptor blocker; CI: confidence interval; DB: direct bilirubin; eGFR: estimated glomerular filtration rate; LAD: left atrial diameter; LVEDD: left ventricular end-diastolic dimension; LVEF: left ventricular ejection fraction; NYHA: New York Heart Association; OR: odds ratio; TB: total bilirubin.

After a median 27 months of follow-up, 146 (14.2%) patients died. The Kaplan–Meier curve indicated that the cumulative rate of long-term survival rate was significantly lower in patients with higher ALBI grade (log-rank test = 45.50, p < 0.001, Figure 1). The univariate Cox proportional hazard model of long-term mortality is shown in Table 3. ALBI score was associated with increased risk of long-term death (unadjusted hazard ratio = 3.16, 95% CI: 2.31 – 4.33, p < 0.001). Other significant variables included age, NYHA functional class > II, anemia, eGFR < 60 mL/min/1.73 m^2^, lgALT, hypoproteinemia, total bilirubin, LAD, LVEDD, LVEF, and β-blocker and digoxin use. These significant risk factors, except the components of ALBI score, were included in the multivariate Cox survival model, which revealed that ALBI score remained an independent predictor for long-term mortality (adjusted hazard ratio = 2.84, 95% CI: 1.95 – 4.13, p < 0.001, Table 4). In addition, the ALBI score was included in this model as a categorical variable rather than a continuous one. The result showed that, compared with ALBI grade 1, the adjusted hazard ratio was 5.69 (95% CI: 1.40 – 23.18, p = 0.015, Table 4) and 16.79 (95% CI: 3.91 – 72.04, p < 0.001, Table 4) for Grade 2 and 3, respectively.

**Figure 1:** Kaplan–Meier curve of overall survival.

**Table 3:** Univariate Cox proportional hazard of long-term mortality

Clinical variables	HR	95% CI	p-value
ALBI score	3.16	2.31 – 4.33	<0.001
Age (years)	1.03	1.02 – 1.04	<0.001
Female sex	0.96	0.67 – 1.39	0.845
Hypertension	0.99	0.69 – 1.44	0.975
Diabetes	0.96	0.62 – 1.49	0.854
Smokers	1.03	0.72 – 1.49	0.859
NYHA functional class>II	1.81	1.28 – 2.54	0.001
Anemia	1.97	1.25 – 3.10	0.003
eGFR<60mL/min/1.73 m^2^	2.09	1.51 – 2.91	<0.001
lgALT	1.78	1.21 – 2.62	0.004
Hypoproteinemia	2.46	1.73 – 3.48	<0.001
Total bilirubin	1.01	1.00 – 1.01	<0.001
LAD	1.03	1.01 – 1.05	0.016
LVEDD	1.04	1.03 – 1.06	<0.001
LVEF	0.96	0.94 – 0.98	<0.001
ACEI/ARB usage	0.93	0.60 – 1.44	0.733
Beta-blocker usage	0.53	0.37 – 0.75	<0.001
Lasix usage	1.08	0.67 – 1.76	0.742
Aldactone usage	1.43	0.83 – 2.48	0.202
Digoxin usage	1.55	1.09 – 2.20	0.016

ACEI: angiotensin-converting enzyme inhibitors; ALBI: albumin-bilirubin; ARB: angiotensin receptor blocker; ALT: alanine transaminase; CI: confidence interval; eGFR: estimated glomerular filtration rate; HR: hazard ratio; LAD: left atrial diameter; LVEDD: left ventricular end-diastolic dimension; LVEF: left ventricular ejection fraction; NYHA: New York Heart Association.

**Table 4:** Multivariate Cox proportional hazard of long-term mortality

Clinical variables		HR	95% CI	p-value
**Model 1**				
ALBI		2.84	1.95 – 4.13	<0.001
Age (years)		1.03	1.02 – 1.05	<0.001
NYHA functional class>II		1.25	0.86 – 1.82	0.236
Anemia		1.25	0.76 – 2.06	0.382
eGFR<60mL/min/1.73 m^2^		1.30	0.91 – 1.85	0.156
lgALT		1.46	1.00 – 2.14	0.050
LAD		1.00	0.98 – 1.03	0.898
LVEDD		1.04	1.02 – 1.06	<0.001
LVEF		0.99	0.97 – 1.01	0.348
Beta-blocker usage		0.65	0.45 – 0.95	0.024
Digoxin usage		1.05	0.72 – 1.54	0.804
**Model 2**				
ALBI				
	Grade 1	-	-	-
	Grade 2	5.69	1.40 – 23.18	0.015
	Grade 3	16.79	3.91 – 72.04	<0.001
Age (years)		1.03	1.02 – 1.05	<0.001
NYHA functional class>II		1.24	0.85 – 1.81	0.262
Anemia		1.37	0.84 – 2.24	0.205
eGFR<60mL/min/1.73 m^2^		1.29	0.90 – 1.84	0.168
lgALT		1.57	1.08 – 2.28	0.019
LAD		1.00	0.98 – 1.03	0.758
LVEDD		1.04	1.02 – 1.07	<0.001
LVEF		0.98	0.96 – 1.01	0.180
Beta-blocker usage		0.59	0.41 – 0.85	0.005
Digoxin usage		1.08	0.74 – 1.58	0.702

ALBI: albumin-bilirubin; ALT: alanine transaminase; CI: confidence interval; eGFR: estimated glomerular filtration rate; HR: hazard ratio; LAD: left atrial diameter; LVEDD: left ventricular end-diastolic dimension; LVEF: left ventricular ejection fraction; NYHA: New York Heart Association.

ROC curve analysis indicated that the area under the curve of ALBI score, serum albumin, and total bilirubin for predicting long-term death were 0.684 (95% CI: 0.654 – 0.714, Figure 2), 0.662 (95% CI: 0.631 – 0.692, Figure 2) and 0.588 (95% CI: 0.556 – 0.619, Figure 2) respectively. ALBI score exhibited relatively superior predictive ability for long-term death than serum albumin (0.684 versus 0.662, p = 0.026, Figure 2) and total bilirubin (0.684 versus 0.588, p = 0.002, Figure 2).

**Figure 2:** ROC analysis of long-term mortality.

## Discussion

To our knowledge, this is the first study to evaluate the prognostic role of ALBI score in patients with idiopathic DCM. The results showed that ALBI score was an independent risk factor for in-hospital MACEs and long-term mortality. In addition, ALBI score exhibited better predictive ability for long-term death than serum albumin and total bilirubin. The ALBI score can be easily measured and would be useful in identifying idiopathic DCM patients who are at a high risk of poor outcomes.

DCM is characterized by ventricular remodeling which can gradually develop into left heart failure and even global heart failure.^13,14^ In addition, right ventricular dysfunction is prevalent in patients with DCM, ^15^ and it has been demonstrated to influence the course and prognosis of DCM.^16^ Progression of right ventricular dysfunction can lead to systemic congestion, resulting in sinusoidal congestion and peri-sinusoidal edema, which impair delivery of oxygen and nutrients to hepatocytes.^17-19^ In addition, decreased cardiac output and inadequate liver perfusion may trigger hypoxic injury. This injury of hepatocytes can manifest as decreased serum albumin and elevated bilirubin.

Albumin, which reflects the synthetic function of the liver, has multiple physiological roles, such as counterbalancing hydrostatic pressure, antioxidant and anti-inflammatory functions, and transporting molecules and drugs.^20^ We found that hypoalbuminemia was related to adverse outcome in patients with idiopathic DCM. This could be explained by several theories. First, in addition to being a marker of liver injury, hypoalbuminemia is frequently associated with renal dysfunction.^20,21^ Albumin is restricted by the normal glomerular barrier, and filtered albumin can be reabsorbed by proximal tubular cells.^22^ However, increased protein urine discharge can be found in renal insufficiency, which results in hypoalbuminemia. Therefore, hypoalbuminemia might reflect the concurrent renal dysfunction and portend poor outcomes. Second, hypoalbuminemia results in lower serum osmotic pressure and can exacerbate pulmonary edema and pleural effusion, precipitating refractory heart failure in patients with DCM.^21^ Third, serum albumin and prealbumin levels have been shown to reflect nutritional status.^23,24^ Malnutrition at times may progress to cardiac cachexia, which is characterized by protein-calorie malnutrition with muscle wasting and peripheral edema, leading to poor quality of life and increased mortality.^24^

Similarly, in patients with advanced DCM, several metabolic processes of bilirubin in the liver, including uptake, conjugation, and secretion, are attenuated by hepatocellular hypoxia and congestion, leading to greater increase in serum total bilirubin. Although bilirubin has antioxidant and anti-inflammatory properties, extremely elevated bilirubin levels represent impaired hemodynamics caused by right ventricular dysfunction, which has an adverse prognostic effect on patients with DCM.^16^ In addition, hyperbilirubinemia reflects poor latent cardiac status in chronic heart failure.^25^ Lang et al. indicated that bilirubin had adverse effects on erythrocytes, inducing suicidal death of erythrocytes. Excessive damage to erythrocytes leads to severe anemia and further affects the prognosis.^26^ These pieces of evidence support our finding that hyperbilirubinemia is a risk factor for patients with idiopathic DCM.

Both hypoalbuminemia and hyperbilirubinemia were risk factors for poor prognosis in patients with idiopathic DCM. The ALBI score, combining these two effects, has been extensively tested as an objective, simple, and distinguishing method for assessing liver function.^27^ To the best of our knowledge, no study yet has evaluated the prognostic value of ALBI score in patients with idiopathic DCM. The present study demonstrated that ALBI score was independently associated with in-hospital and long-term adverse outcomes. The ALBI score consists of only two variables, and it is a simple risk-stratifying tool in patients with idiopathic DCM. Based on the current study, the clinical application of the ALBI score might be extended from hepatic diseases to idiopathic DCM.

### Limitations

Our study has some limitations. First, this was a retrospective cohort study; therefore, some admission bilirubin and albumin levels were missing, which may affect the results. Second, bilirubin and albumin were not dynamically detected. The relationship between prognosis and ALBI score at different time points is unknown. Finally, as our included study population did not represent patients with idiopathic DCM in different settings, such as in western countries, the study results should be validated in different idiopathic DCM cohorts.

## Conclusions

This study showed that the ALBI score was independently associated with increased risk of in-hospital MACEs and long-term mortality in patients with idiopathic DCM. Moreover, compared to bilirubin and albumin, the ALBI score exhibited relatively superior predictive ability for long-term mortality, which might identify more patients at high risk of poor outcomes.
